# {*N*,*N*-Dimethyl-*N*′-[1-(2-pyrid­yl)ethyl­idene]propane-1,3-diamine}bis(thio­cyanato-κ*N*)­copper(II)

**DOI:** 10.1107/S1600536810036378

**Published:** 2010-09-18

**Authors:** Ling-Wei Xue, Gan-Qing Zhao, Yong-Jun Han, Li-Hua Chen, Qin-Long Peng

**Affiliations:** a College of Chemistry and Chemical Engineering, Pingdingshan University, Pingdingshan, Henan 467000, People’s Republic of China

## Abstract

In the title complex, [Cu(NCS)_2_(C_12_H_19_N_3_)], the Cu^II^ atom is five-coordinated in a square-pyramidal geometry defined by one pyridine N, one imine N, and one amine N atom of the tridentate Schiff base ligand and two N-bonded thio­cyanate ions (one of the latter occupying the apical site). The three bridging C atoms and the two terminal C atoms of the Schiff base are disordered over two sets of sites, with occupancies of 0.465 (2) and 0.535 (2).

## Related literature

For a related structure and background to Schiff bases, see: Xue *et al.* (2010 ▶).
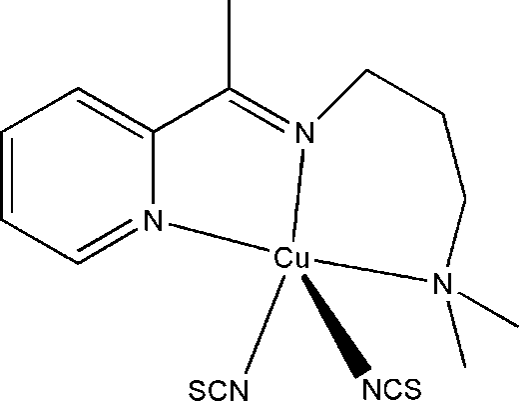


## Experimental

### 

#### Crystal data

[Cu(NCS)_2_(C_12_H_19_N_3_)]
*M* *_r_* = 385.00
Monoclinic, 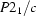

*a* = 13.723 (2) Å
*b* = 7.2380 (12) Å
*c* = 18.237 (3) Å
β = 103.559 (2)°
*V* = 1760.9 (5) Å^3^
*Z* = 4
Mo *K*α radiation
μ = 1.48 mm^−1^
*T* = 298 K
0.23 × 0.21 × 0.21 mm

#### Data collection

Bruker SMART CCD diffractometer
Absorption correction: multi-scan (*SADABS*; Sheldrick, 1996 ▶) *T*_min_ = 0.727, *T*_max_ = 0.746
13886 measured reflections
3816 independent reflections
2698 reflections with *I* > 2σ(*I*)
*R*_int_ = 0.041

#### Refinement

*R*[*F* ^2^ > 2σ(*F* ^2^)] = 0.042
*wR*(*F* ^2^) = 0.111
*S* = 1.04
3816 reflections
237 parameters
16 restraints
H-atom parameters constrained
Δρ_max_ = 0.57 e Å^−3^
Δρ_min_ = −0.47 e Å^−3^

### 

Data collection: *SMART* (Bruker, 1998 ▶); cell refinement: *SAINT* (Bruker, 1998 ▶); data reduction: *SAINT*; program(s) used to solve structure: *SHELXS97* (Sheldrick, 2008 ▶); program(s) used to refine structure: *SHELXL97* (Sheldrick, 2008 ▶); molecular graphics: *SHELXTL* (Sheldrick, 2008 ▶); software used to prepare material for publication: *SHELXTL*.

## Supplementary Material

Crystal structure: contains datablocks global, I. DOI: 10.1107/S1600536810036378/hb5636sup1.cif

Structure factors: contains datablocks I. DOI: 10.1107/S1600536810036378/hb5636Isup2.hkl

Additional supplementary materials:  crystallographic information; 3D view; checkCIF report

## Figures and Tables

**Table 1:** Selected bond lengths (Å)

Cu1—N5	1.955 (3)
Cu1—N2	2.013 (3)
Cu1—N1	2.047 (3)
Cu1—N3	2.078 (3)
Cu1—N4	2.153 (3)

**Fig. 1.**
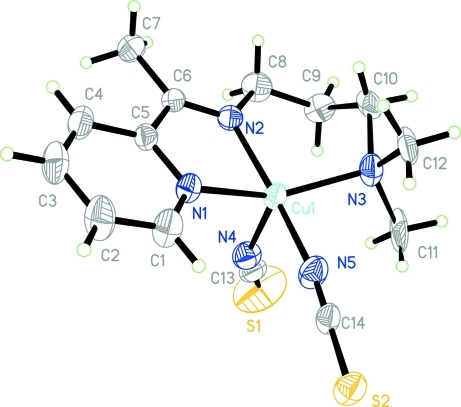
The structure of the title complex with 30% probability displacement ellipsoids.

## supplementary crystallographic information

### Comment

Recently, we have reported a copper(II) complex with a Schiff base ligand (Xue *et al.*, 2010). In this paper, a new thiocyanato-coordinated mononuclear copper(II) complex with the Schiff base *N,N*-dimethyl-*N'*-(1-pyridin-2-ylethylidene)propane-1,3-diamine, is reported.

The Cu atom in the complex, Fig. 1, is five-coordinate in a square pyramidal geometry, with one pyridine N, one imine N, and one amine N atoms of a Schiff base ligand, and with one thiocyanate N atom, occupying the basal plane, and with another thiocyanate N atom occupying the apical position. The Cu atom displaced 0.306 (2) Å from the plane defined by the four basal donor atoms. The slight distortion of the square pyramidal coordination can be observed from the coordinate bond lengths and angles (Table 1).

### Experimental

2-Acetylpyridine (121 mg, 1.0 mmol), *N,N*dimethylpropane-1,3-diamine (102 mg, 1.0 mmol), ammonium thiocyanate (76 mg, 1.0 mmol), and copper acetate monohydrate (199.2 mg, 1.0 mmol) were dissolved in methanol (80 ml). The mixture was stirred for two hours at room temperature. The resulting solution was left in air for a few days, yielding blue blocks of (I).

### Refinement

H atoms were placed in idealized positions and constrained to ride on their parent atoms with C—H distances of 0.93–0.97 Å, and with *U*_iso_(H) set at 1.2*U*_eq_(C) and 1.5*U*_eq_(C_methyl_). The three briding C atoms and the two terminal C atoms of the Schiff base ligand are disordered over two sites, with occupancies of 0.465 (2) and 0.535 (2).

### Crystal data

**Table:**

[Cu(NCS)_2_(C_12_H_19_N_3_)]	*F*(000) = 796
*M**_r_* = 385.00	*D*_x_ = 1.452 Mg m^−^^3^
Monoclinic, *P*2_1_/*c*	Mo *K*α radiation, λ = 0.71073 Å
Hall symbol: -P 2ybc	Cell parameters from 2747 reflections
*a* = 13.723 (2) Å	θ = 2.3–24.5°
*b* = 7.2380 (12) Å	µ = 1.48 mm^−^^1^
*c* = 18.237 (3) Å	*T* = 298 K
β = 103.559 (2)°	Block, blue
*V* = 1760.9 (5) Å^3^	0.23 × 0.21 × 0.21 mm
*Z* = 4	

### Data collection

**Table:**

Bruker SMART CCD diffractometer	3816 independent reflections
Radiation source: fine-focus sealed tube	2698 reflections with *I* > 2σ(*I*)
graphite	*R*_int_ = 0.041
ω scans	θ_max_ = 27.0°, θ_min_ = 2.3°
Absorption correction: multi-scan (*SADABS*; Sheldrick, 1996)	*h* = −15→17
*T*_min_ = 0.727, *T*_max_ = 0.746	*k* = −9→9
13886 measured reflections	*l* = −23→23

### Refinement

**Table:**

Refinement on *F*^2^	Primary atom site location: structure-invariant direct methods
Least-squares matrix: full	Secondary atom site location: difference Fourier map
*R*[*F*^2^ > 2σ(*F*^2^)] = 0.042	Hydrogen site location: inferred from neighbouring sites
*wR*(*F*^2^) = 0.111	H-atom parameters constrained
*S* = 1.03	*w* = 1/[σ^2^(*F*_o_^2^) + (0.0492*P*)^2^ + 0.6842*P*] where *P* = (*F*_o_^2^ + 2*F*_c_^2^)/3
3816 reflections	(Δ/σ)_max_ < 0.001
237 parameters	Δρ_max_ = 0.57 e Å^−^^3^
16 restraints	Δρ_min_ = −0.47 e Å^−^^3^

### Special details

**Table:**

Geometry. All e.s.d.'s (except the e.s.d. in the dihedral angle between two l.s. planes) are estimated using the full covariance matrix. The cell e.s.d.'s are taken into account individually in the estimation of e.s.d.'s in distances, angles and torsion angles; correlations between e.s.d.'s in cell parameters are only used when they are defined by crystal symmetry. An approximate (isotropic) treatment of cell e.s.d.'s is used for estimating e.s.d.'s involving l.s. planes.
Refinement. Refinement of *F*^2^ against ALL reflections. The weighted *R*-factor *wR* and goodness of fit *S* are based on *F*^2^, conventional *R*-factors *R* are based on *F*, with *F* set to zero for negative *F*^2^. The threshold expression of *F*^2^ > σ(*F*^2^) is used only for calculating *R*-factors(gt) *etc*. and is not relevant to the choice of reflections for refinement. *R*-factors based on *F*^2^ are statistically about twice as large as those based on *F*, and *R*- factors based on ALL data will be even larger.

### Fractional atomic coordinates and isotropic or equivalent isotropic displacement parameters (Å^2^)

**Table:**

	*x*	*y*	*z*	*U*_iso_*/*U*_eq_	Occ. (<1)
Cu1	0.24814 (3)	0.82783 (5)	0.39009 (2)	0.04378 (15)	
N1	0.3853 (2)	0.8651 (4)	0.36614 (15)	0.0459 (7)	
N2	0.2846 (2)	1.0789 (4)	0.43585 (14)	0.0457 (6)	
N4	0.2763 (2)	0.7053 (4)	0.50065 (18)	0.0594 (8)	
N5	0.2289 (2)	0.5969 (5)	0.33292 (18)	0.0624 (8)	
S1	0.2253 (2)	0.6298 (3)	0.63328 (10)	0.1725 (10)	
S2	0.23557 (7)	0.28351 (14)	0.24673 (6)	0.0611 (3)	
C1	0.4337 (3)	0.7542 (6)	0.3285 (2)	0.0647 (10)	
H1	0.4028	0.6446	0.3090	0.078*	
C2	0.5270 (3)	0.7936 (7)	0.3170 (2)	0.0752 (13)	
H2	0.5584	0.7124	0.2904	0.090*	
C3	0.5724 (3)	0.9539 (7)	0.3454 (2)	0.0716 (12)	
H3	0.6358	0.9835	0.3388	0.086*	
C4	0.5236 (3)	1.0714 (6)	0.3839 (2)	0.0601 (10)	
H4	0.5533	1.1821	0.4032	0.072*	
C5	0.4300 (2)	1.0238 (5)	0.39356 (17)	0.0443 (7)	
C6	0.3702 (2)	1.1400 (4)	0.43399 (18)	0.0452 (8)	
C7	0.4155 (3)	1.3171 (5)	0.4690 (2)	0.0701 (12)	
H7A	0.4090	1.4104	0.4307	0.105*	
H7B	0.4851	1.2979	0.4921	0.105*	
H7C	0.3812	1.3563	0.5065	0.105*	
N3	0.0939 (2)	0.8665 (4)	0.36804 (17)	0.0606 (8)	0.465 (11)
C8	0.2202 (3)	1.1843 (5)	0.4750 (2)	0.0684 (11)	0.465 (11)
H8A	0.2116	1.3077	0.4538	0.082*	0.465 (11)
H8B	0.2549	1.1961	0.5276	0.082*	0.465 (11)
C9	0.1197 (6)	1.1045 (16)	0.4713 (5)	0.062 (3)	0.465 (11)
H9A	0.0797	1.1912	0.4924	0.074*	0.465 (11)
H9B	0.1263	0.9918	0.5009	0.074*	0.465 (11)
C10	0.0677 (7)	1.0625 (13)	0.3896 (5)	0.056 (3)	0.465 (11)
H10A	0.0886	1.1517	0.3566	0.067*	0.465 (11)
H10B	−0.0043	1.0731	0.3830	0.067*	0.465 (11)
C11	0.053 (3)	0.696 (4)	0.396 (2)	0.092 (10)	0.465 (11)
H11A	−0.0185	0.7058	0.3873	0.138*	0.465 (11)
H11B	0.0812	0.6838	0.4496	0.138*	0.465 (11)
H11C	0.0705	0.5903	0.3705	0.138*	0.465 (11)
C12	0.066 (3)	0.888 (6)	0.2842 (6)	0.071 (8)	0.465 (11)
H12A	−0.0053	0.9054	0.2678	0.107*	0.465 (11)
H12B	0.0849	0.7789	0.2611	0.107*	0.465 (11)
H12C	0.0998	0.9931	0.2700	0.107*	0.465 (11)
N3'	0.0939 (2)	0.8665 (4)	0.36804 (17)	0.0606 (8)	0.535 (11)
C8'	0.2202 (3)	1.1843 (5)	0.4750 (2)	0.0684 (11)	0.535 (11)
H8'A	0.2415	1.3124	0.4803	0.082*	0.535 (11)
H8'B	0.2252	1.1336	0.5250	0.082*	0.535 (11)
C9'	0.1099 (5)	1.1713 (11)	0.4277 (7)	0.077 (3)	0.535 (11)
H9'A	0.0703	1.2595	0.4484	0.093*	0.535 (11)
H9'B	0.1091	1.2112	0.3768	0.093*	0.535 (11)
C10'	0.0573 (6)	0.9862 (12)	0.4224 (6)	0.069 (3)	0.535 (11)
H10C	0.0711	0.9278	0.4717	0.082*	0.535 (11)
H10D	−0.0145	1.0035	0.4055	0.082*	0.535 (11)
C11'	0.036 (2)	0.703 (3)	0.3837 (17)	0.071 (5)	0.535 (11)
H11D	−0.0346	0.7331	0.3720	0.106*	0.535 (11)
H11E	0.0569	0.6702	0.4360	0.106*	0.535 (11)
H11F	0.0467	0.6009	0.3531	0.106*	0.535 (11)
C12'	0.052 (3)	0.896 (5)	0.2860 (6)	0.071 (7)	0.535 (11)
H12D	−0.0197	0.9133	0.2770	0.106*	0.535 (11)
H12E	0.0653	0.7900	0.2584	0.106*	0.535 (11)
H12F	0.0815	1.0035	0.2697	0.106*	0.535 (11)
C13	0.2546 (3)	0.6730 (5)	0.5551 (2)	0.0572 (9)	
C14	0.2316 (2)	0.4674 (5)	0.29685 (19)	0.0481 (8)	

### Atomic displacement parameters (Å^2^)

**Table:**

	*U*^11^	*U*^22^	*U*^33^	*U*^12^	*U*^13^	*U*^23^
Cu1	0.0369 (2)	0.0457 (2)	0.0496 (2)	−0.00511 (18)	0.01183 (16)	−0.00953 (18)
N1	0.0412 (15)	0.0515 (17)	0.0463 (15)	−0.0058 (12)	0.0131 (12)	−0.0059 (12)
N2	0.0448 (16)	0.0426 (16)	0.0496 (15)	0.0014 (13)	0.0109 (12)	−0.0037 (12)
N4	0.066 (2)	0.057 (2)	0.0533 (18)	−0.0049 (15)	0.0097 (15)	0.0003 (15)
N5	0.0559 (19)	0.0598 (19)	0.072 (2)	−0.0085 (16)	0.0162 (16)	−0.0233 (17)
S1	0.264 (3)	0.178 (2)	0.1026 (12)	−0.0650 (18)	0.0974 (15)	0.0160 (12)
S2	0.0560 (6)	0.0568 (6)	0.0762 (6)	−0.0069 (4)	0.0269 (5)	−0.0187 (5)
C1	0.055 (2)	0.078 (3)	0.064 (2)	−0.011 (2)	0.0212 (19)	−0.025 (2)
C2	0.051 (2)	0.107 (4)	0.075 (3)	−0.005 (2)	0.031 (2)	−0.023 (2)
C3	0.042 (2)	0.106 (4)	0.071 (3)	−0.012 (2)	0.0205 (19)	0.002 (2)
C4	0.045 (2)	0.069 (3)	0.064 (2)	−0.0139 (19)	0.0068 (17)	0.003 (2)
C5	0.0366 (17)	0.0494 (19)	0.0435 (17)	−0.0030 (15)	0.0024 (13)	0.0060 (15)
C6	0.0428 (19)	0.0389 (18)	0.0486 (18)	−0.0020 (14)	0.0004 (14)	0.0025 (14)
C7	0.062 (2)	0.046 (2)	0.093 (3)	−0.0065 (19)	−0.001 (2)	−0.012 (2)
N3	0.0378 (16)	0.073 (2)	0.069 (2)	−0.0027 (15)	0.0079 (14)	−0.0122 (17)
C8	0.069 (3)	0.059 (2)	0.079 (3)	0.009 (2)	0.023 (2)	−0.021 (2)
C9	0.062 (6)	0.061 (7)	0.078 (7)	0.003 (5)	0.047 (5)	−0.009 (5)
C10	0.034 (5)	0.066 (7)	0.072 (6)	0.017 (4)	0.019 (4)	0.007 (5)
C11	0.019 (8)	0.155 (18)	0.096 (19)	−0.026 (9)	0.003 (9)	0.037 (10)
C12	0.032 (9)	0.105 (17)	0.065 (12)	−0.007 (8)	−0.012 (8)	0.027 (10)
N3'	0.0378 (16)	0.073 (2)	0.069 (2)	−0.0027 (15)	0.0079 (14)	−0.0122 (17)
C8'	0.069 (3)	0.059 (2)	0.079 (3)	0.009 (2)	0.023 (2)	−0.021 (2)
C9'	0.067 (6)	0.058 (6)	0.126 (10)	0.003 (4)	0.061 (7)	−0.008 (6)
C10'	0.058 (5)	0.074 (6)	0.083 (7)	0.005 (4)	0.034 (5)	0.001 (5)
C11'	0.038 (12)	0.120 (12)	0.053 (6)	−0.007 (6)	0.007 (8)	0.019 (6)
C12'	0.050 (13)	0.061 (10)	0.100 (15)	−0.006 (7)	0.013 (8)	0.010 (9)
C13	0.066 (2)	0.043 (2)	0.061 (2)	−0.0099 (18)	0.0108 (19)	0.0024 (18)
C14	0.0348 (17)	0.054 (2)	0.0550 (19)	−0.0070 (15)	0.0103 (14)	−0.0051 (17)

### Geometric parameters (Å, °)

**Table:**

Cu1—N5	1.955 (3)	N3—C11	1.494 (9)
Cu1—N2	2.013 (3)	N3—C10	1.537 (7)
Cu1—N1	2.047 (3)	C8—C9	1.483 (7)
Cu1—N3	2.078 (3)	C8—H8A	0.9700
Cu1—N4	2.153 (3)	C8—H8B	0.9700
N1—C1	1.331 (4)	C9—C10	1.525 (9)
N1—C5	1.343 (4)	C9—H9A	0.9700
N2—C6	1.264 (4)	C9—H9B	0.9700
N2—C8	1.472 (4)	C10—H10A	0.9700
N4—C13	1.126 (4)	C10—H10B	0.9700
N5—C14	1.151 (4)	C11—H11A	0.9600
S1—C13	1.600 (4)	C11—H11B	0.9600
S2—C14	1.622 (4)	C11—H11C	0.9600
C1—C2	1.375 (5)	C12—H12A	0.9600
C1—H1	0.9300	C12—H12B	0.9600
C2—C3	1.361 (6)	C12—H12C	0.9600
C2—H2	0.9300	C9'—C10'	1.514 (9)
C3—C4	1.373 (5)	C9'—H9'A	0.9700
C3—H3	0.9300	C9'—H9'B	0.9700
C4—C5	1.380 (5)	C10'—H10C	0.9700
C4—H4	0.9300	C10'—H10D	0.9700
C5—C6	1.486 (5)	C11'—H11D	0.9600
C6—C7	1.500 (4)	C11'—H11E	0.9600
C7—H7A	0.9600	C11'—H11F	0.9600
C7—H7B	0.9600	C12'—H12D	0.9600
C7—H7C	0.9600	C12'—H12E	0.9600
N3—C12	1.494 (9)	C12'—H12F	0.9600
			
N5—Cu1—N2	169.17 (12)	C11—N3—Cu1	105.6 (16)
N5—Cu1—N1	90.84 (12)	C10—N3—Cu1	111.3 (4)
N2—Cu1—N1	79.51 (11)	N2—C8—C9	115.7 (4)
N5—Cu1—N3	90.46 (12)	N2—C8—H8A	108.3
N2—Cu1—N3	95.82 (11)	C9—C8—H8A	108.3
N1—Cu1—N3	152.40 (12)	N2—C8—H8B	108.3
N5—Cu1—N4	96.85 (13)	C9—C8—H8B	108.3
N2—Cu1—N4	90.64 (11)	H8A—C8—H8B	107.4
N1—Cu1—N4	106.39 (12)	C8—C9—C10	109.8 (7)
N3—Cu1—N4	100.82 (12)	C8—C9—H9A	109.7
C1—N1—C5	117.9 (3)	C10—C9—H9A	109.7
C1—N1—Cu1	128.7 (3)	C8—C9—H9B	109.7
C5—N1—Cu1	113.4 (2)	C10—C9—H9B	109.7
C6—N2—C8	119.9 (3)	H9A—C9—H9B	108.2
C6—N2—Cu1	116.6 (2)	C9—C10—N3	110.5 (7)
C8—N2—Cu1	123.3 (2)	C9—C10—H10A	109.5
C13—N4—Cu1	152.2 (3)	N3—C10—H10A	109.5
C14—N5—Cu1	169.4 (3)	C9—C10—H10B	109.5
N1—C1—C2	123.3 (4)	N3—C10—H10B	109.5
N1—C1—H1	118.4	H10A—C10—H10B	108.1
C2—C1—H1	118.4	N3—C11—H11A	109.5
C3—C2—C1	118.6 (4)	N3—C11—H11B	109.5
C3—C2—H2	120.7	H11A—C11—H11B	109.5
C1—C2—H2	120.7	N3—C11—H11C	109.5
C2—C3—C4	119.2 (4)	H11A—C11—H11C	109.5
C2—C3—H3	120.4	H11B—C11—H11C	109.5
C4—C3—H3	120.4	N3—C12—H12A	109.5
C3—C4—C5	119.4 (4)	N3—C12—H12B	109.5
C3—C4—H4	120.3	H12A—C12—H12B	109.5
C5—C4—H4	120.3	N3—C12—H12C	109.5
N1—C5—C4	121.6 (3)	H12A—C12—H12C	109.5
N1—C5—C6	114.3 (3)	H12B—C12—H12C	109.5
C4—C5—C6	124.1 (3)	C10'—C9'—H9'A	107.7
N2—C6—C5	116.1 (3)	C10'—C9'—H9'B	107.7
N2—C6—C7	125.6 (3)	H9'A—C9'—H9'B	107.1
C5—C6—C7	118.3 (3)	C9'—C10'—H10C	109.9
C6—C7—H7A	109.5	C9'—C10'—H10D	109.9
C6—C7—H7B	109.5	H10C—C10'—H10D	108.3
H7A—C7—H7B	109.5	H11D—C11'—H11E	109.5
C6—C7—H7C	109.5	H11D—C11'—H11F	109.5
H7A—C7—H7C	109.5	H11E—C11'—H11F	109.5
H7B—C7—H7C	109.5	H12D—C12'—H12E	109.5
C12—N3—C11	114 (2)	H12D—C12'—H12F	109.5
C12—N3—C10	98.4 (18)	H12E—C12'—H12F	109.5
C11—N3—C10	122.9 (19)	N4—C13—S1	178.9 (4)
C12—N3—Cu1	102.7 (18)	N5—C14—S2	179.4 (3)
